# CircMMP11 overexpression predicts the poor survival of non-small cell lung cancer and downregulates miR-143 through methylation to suppress cell proliferation

**DOI:** 10.1186/s13019-021-01701-w

**Published:** 2021-11-08

**Authors:** Juan Chen, Jiang Gong

**Affiliations:** 1 Department of Pathology, Bishan People’s Hospital of Chongqing, Chongqing, 402760 People’s Republic of China; 2 Department of Laboratory Medicine, Wanzhou People’s Hospital of Chongqing, No. 27 Guoben Road, Wanzhou District, Chongqing, 404100 People’s Republic of China

**Keywords:** Non-small cell lung cancer, circMMP11, miR-143, Methylation

## Abstract

**Background:**

CircMMP11 is a characterized circRNA with oncogenic function in breast cancer. In this study, we explored the involvement of circMMP11 in non-small cell lung cancer (NSCLC).

**Methods:**

Paired cancer and non-cancer tissues were collected from 66 NSCLC patients, and the expression of circMMP11 and miR-143 in these tissues were detected using RT-qPCRs. Overexpression levels of circMMP11 and miR-143 were performed by transfection, and their crosstalk was analyzed by RT-qPCRs. The effect of circMMP11 overexpression on miR-143 methylation was analyzed by methylation-specific PCR. CCK-8 assay was performed to analyze the roles of miR-143 and circMMP11 in regulating NSCLC cell proliferation.

**Results:**

We found that circMMP11 was overexpressed in NSCLC and predicted patients’ poor survival. Moreover, a close correlation between circMMP11 and miR143 was observed. In NSCLC cells, circMMP11 overexpression reduced miR-143 expression and increased miR-143 methylation. CCK-8 assay analysis showed that miR-143 reversed the enhancing effects of circMMP11 overexpression on cell proliferation.

**Conclusions:**

CircMMP11 is overexpressed in NSCLC and predicts poor survival. In addition, circMMP11 may downregulate miR-143 through methylation to suppress cell proliferation.

## Background

Non-small cell lung cancer (NSCLC) is the major subtype of lung cancer and accounts for more than 80% of all cases [1]. NSCLC is also the leading cause of death among both male and female cancer patients [2, 3]. The major challenge in the treatment of NSCLC is tumor metastasis [2, 3]. It is estimated that more than 61% of NSCLC patients with localized disease can survive 5 years. However, more than 40% of NSCLC patients are diagnosed with metastatic tumors. Once distant tumor metastasis occurs, the overall 5-year survival rate will drop to below 10% [4, 5]. Therefore, developing more effective diagnostic and therapeutic approaches is needed.

Most NSCLC cases are caused by smoking [6]. However, NSCLC can also affect never-smokers [7], indicating the involvement of other factors, such as molecular factors [8–10]. Some molecular players have been characterized as potential targets for treating NSCLC [8–10]. For instance, anaplastic lymphoma kinase (ALK) inhibitors have been widely used to suppress NSCLC progression in clinical trials [8]. However, molecular targeted therapy is still under research, and more targets are needed. Circular RNAs (circRNAs) are covalently closed RNA transcripts that regulate gene expression to participate in cancers rather than coding proteins [11, 12]. Some circRNAs have been recognized as potential targets for targeted cancer therapy [13]. However, the functions of most circRNAs in cancer biology are unknown. CircMMP11 is a characterized oncogenic circRNA in breast cancer [14]. Our preliminary deep sequencing analysis showed that circMMP11 is overexpressed in NSCLC and is inversely correlated with miR-143, which also participates in cancer biology [15]. Therefore, we further analyzed the interaction between circMMP11 and miR-143 in NSCLC.

## Methods

### Research subjects

This study enrolled a total of 66 NSCLC patients (38 males and 28 females, 52.4 ± 5.7 years) at Wanzhou People’s Hospital of Chongqing from June 2013 to August 2015. This study was approved by the Ethics Committee of this hospital. Because other clinical disorders and therapies may also disturb gene expression, patients complicated with other clinical disorders and received any therapies with 3 months prior to admission were excluded. Patients with a history of any malignancies were also excluded. All patients signed informed consent.

### A 5-year follow-up

According to the AJCC system, the 66 patients included 20 cases at stage I or II, and 46 cases at stage III or IV. Patients were treated with surgical resection, radiation therapy, chemical drugs, or a combination of these therapies. Therapies were determined mainly based on the clinical stage. The 66 patients were visited every month for 5 years to monitor their survival conditions. All 66 patients completed the follow-up.

### Tissue acquisition and cell lines

The collection of cancer and non-cancer tissue samples from each patient was performed through fine needle aspiration. Histopathological analysis was performed to confirm all tissue samples. Tissue samples (fresh) were kept in liquid nitrogen prior to the subsequent assays.

The NSCLC cell models used in this study were Calu-3 and H2170 cell lines from ATCC (USA). Both cell lines were cultured in media composed of 10% FBS and 90% RPMI-1640 medium at 37 °C in an incubator with 95% humidity and 5% CO_2_.

### Cell transfections

CircMMP11 and miR-143 were overexpressed in both Calu-3 and H2170 cells by transfecting circMMP11 expression vector or miR-143 mimic into 10^8^ cells using Lipofectamine 2000 (Invitrogen). The construction of circMMP11 expression vector was performed with pcDNA3.1 vector as the backbone vector (Invitrogen). MiR-143 mimic and negative control (NC) mimic were synthesized by Sangon biotech (Shanghai, China). NC experiments were performed by transfecting NC mimic or empty vector using the same method. Control (C) cells were untransfected cells. A total of 10^6^ cells in each well of a 6-well plate were used for each transfection. Transfected cells were cultured in fresh media for 48 h prior to the subsequent assays.

### Preparation of RNA samples

Total RNAs were extracted from tissue samples and 10^6^ cells using Ribozol (Invitrogen) and treated with DNase I (Invitrogen) at 37 °C for 100 min to remove genomic DNA. RNA purity was examined using OD 260/280 ratios, and a ratio close to 2.0 indicated pure RNA samples. RNA integrity was examined by electrophoresis using 5% urea-PAGE gels.

### RT-qPCR

cDNA samples were prepared using ReverTra Ace™ qPCR RT Master Mix (Toyobo) with RNA samples with satisfactory quality as the templates. CircMMP11 expression was analyzed by qPCRs using SYBR Green Master Mix (Bio-Rad) with 18S rRNA as the endogenous control.

Mature miR-143 levels were analyzed by All-in-One™ miRNA qRT-PCR reagent kit (GeneCopoeia) with U6 as the endogenous control. Primer sequences were 5′-CTAGCTATGCCTACTTCCTGC-3′ (forward) and 5′-CAGAGCCTTCACCTTCAC A-3′ (reverse) for circMMP11; 5′-GTAACCCGTTGAACCCCAT-3′ (forward) and 5′-CCATCCAATCGGTAGTAGC-3′ (reverse) for 18S rRNA; 5′-TGAGATGAAGCACT GTAG-3′ (forward) for miR-143. The above primers were synthesized by Invitrogen (Shanghai, China). Universal reverse primer and U6 forward primer were from the kit.

Ct values of target genes were normalized to corresponding endogenous controls using the 2^−ΔΔCt^ method.

### Methylation-specific PCR (MSP)

Total genomic DNAs were extracted from Calu-3 and H2170 cells with transfections using conventional methods. DNA samples were converted using DNA Methylation-GoldTM kit (ZYMO RESEARCH). After that, miR-143 gene methylation was analyzed by MSPs and routine PCRs using 2 × Taq mixture (Invitrogen).

### Cell counting kit-8 (CCK-8) assay

Calu-3 and H2170 cells with transfections were subjected to proliferation assay using a CCK-8 assay kit (Sigma-Aldrich). Briefly, cells were cultured in a 96-well cell culture plate with 4000 cells in 0.1 ml medium per well at the methods mentioned above. Each experiment was set in triplicate. CCK-8 solution was added to 10% at 2 h prior to the measurement of OD values at 450 nm, which was performed every 24 h until 96 h.

### Statistical analysis

Differential gene expression in paired tissue samples was presented by heatmaps plotted using Heml 1.0 software. ANOVA Tukey’s test was used to compare independent cell transfection groups. Pearson’s correlation coefficient was used to analyze correlations. The 66 patients were divided into high and low circMMP11 level groups with the medium circMMP11 level in cancer tissues as the cutoff value, and the survival curves were plotted and compared by log-rank test. *P* < 0.05 was statistically significant.

## Results

### CircMMP11 overexpression in NSCLC predicted poor survival

**Fig. 1:**
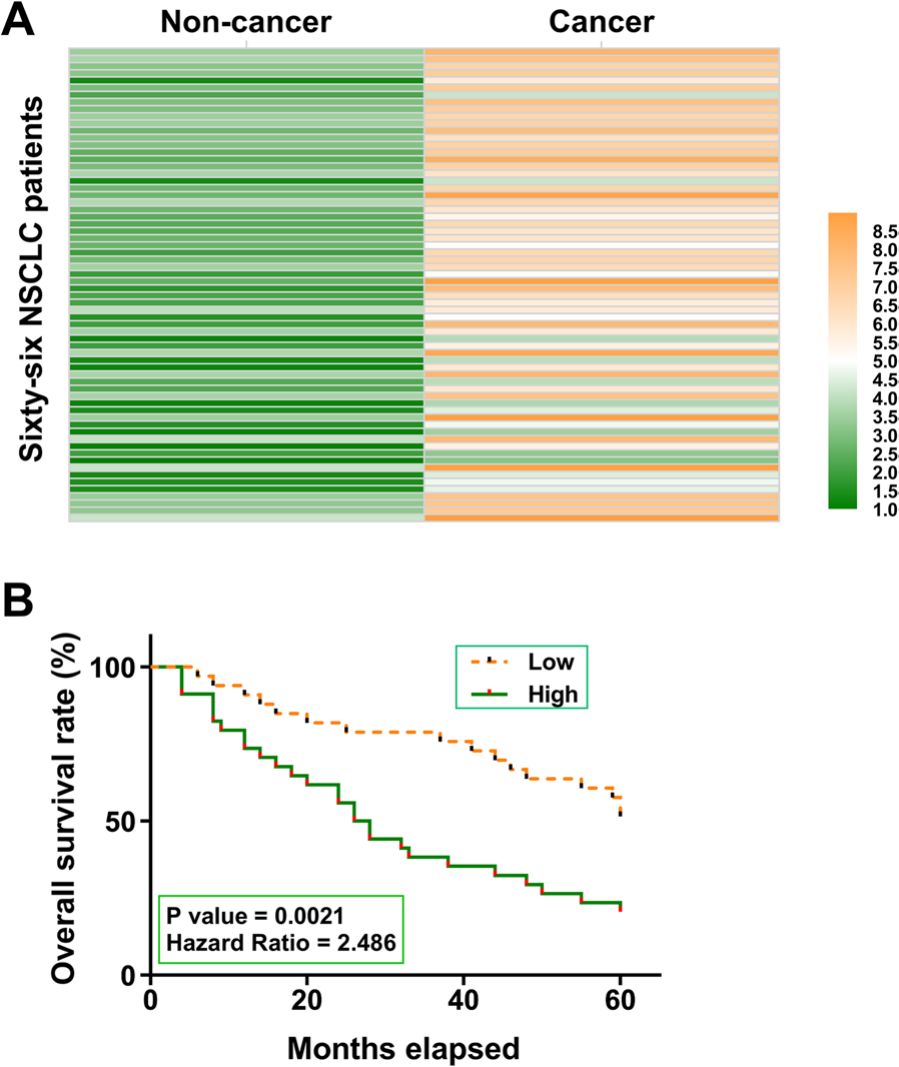
CircMMP11 overexpression in NSCLC predicted poor survival. CircMMP11 expression in paired cancer and non-cancer tissues from the 66 NSCLC patients was analyzed by RT-qPCRs. Differential gene expression in paired tissue samples was presented by heatmaps plotted using Heml 1.0 software (**A**). The 66 patients were divided into high and low circMMP11 level groups (cutoff value = medium expression level in cancer tissues), and the survival curves were plotted and compared by log-rank test (**B**)

CircMMP11 Expression in paired cancer and non-cancer tissues from the 66 NSCLC patients was analyzed by RT-qPCRs. Heatmap analysis showed that circMMP11 expression levels were higher in cancer tissues than in non-cancer tissues (Fig. 1A). Survival analysis showed that patients in the high circMMP11 level group exhibited a significantly higher mortality rate than patients in the low circMMP11 level group during the 5-year follow-up.

### MiR-143 was overexpressed in NSCLC and was inversely correlated with circMMP11

**Fig. 2:**
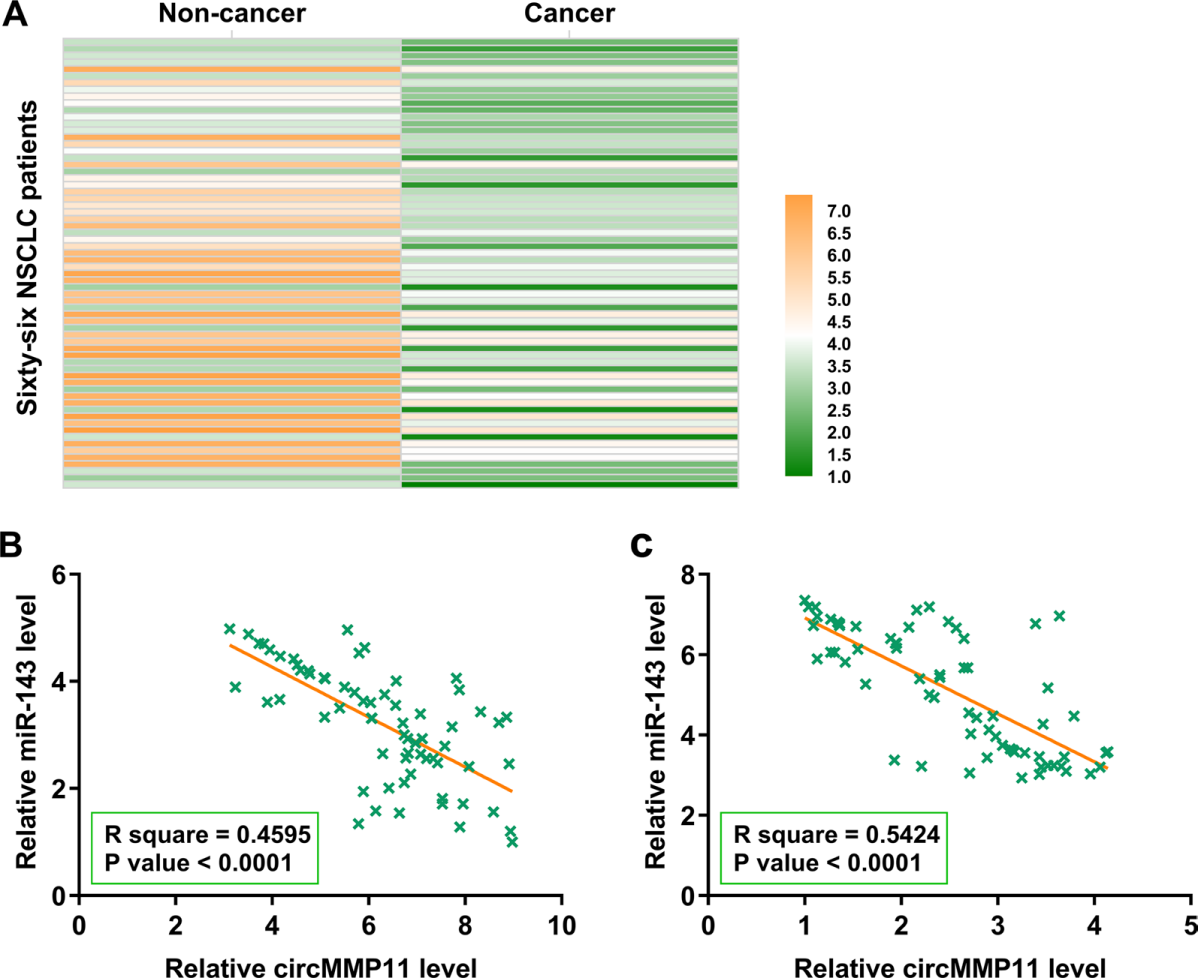
MiR-143 was overexpressed in NSCLC and was inversely correlated with circMMP11. MiR-143 expression in paired cancer and non-cancer tissues from the 66 NSCLC patients was analyzed by RT-qPCRs. Differential gene expression in paired tissue samples was presented by heatmaps plotted using Heml 1.0 software (**A**). Pearson’s correlation coefficient analysis was performed to analyze the correlations between circMMP11 and miR-143 across both cancer (**B**) and non-cancer (**C**) tissue samples

MiR-143 expression in paired cancer and non-cancer tissues from the 66 NSCLC patients was analyzed by RT-qPCRs. Heatmap analysis showed that miR-143 expression levels were lower in cancer tissues than in non-cancer tissues (Fig. 2A). Pearson’s correlation coefficient analysis showed that circMMP11 and miR-143 were inversely and significantly correlated across both cancer (Fig. 2B) and non-cancer (Fig. 2C) tissue samples.

### CircMMP11 overexpression decreased miR-143 expression via methylation

**Fig. 3:**
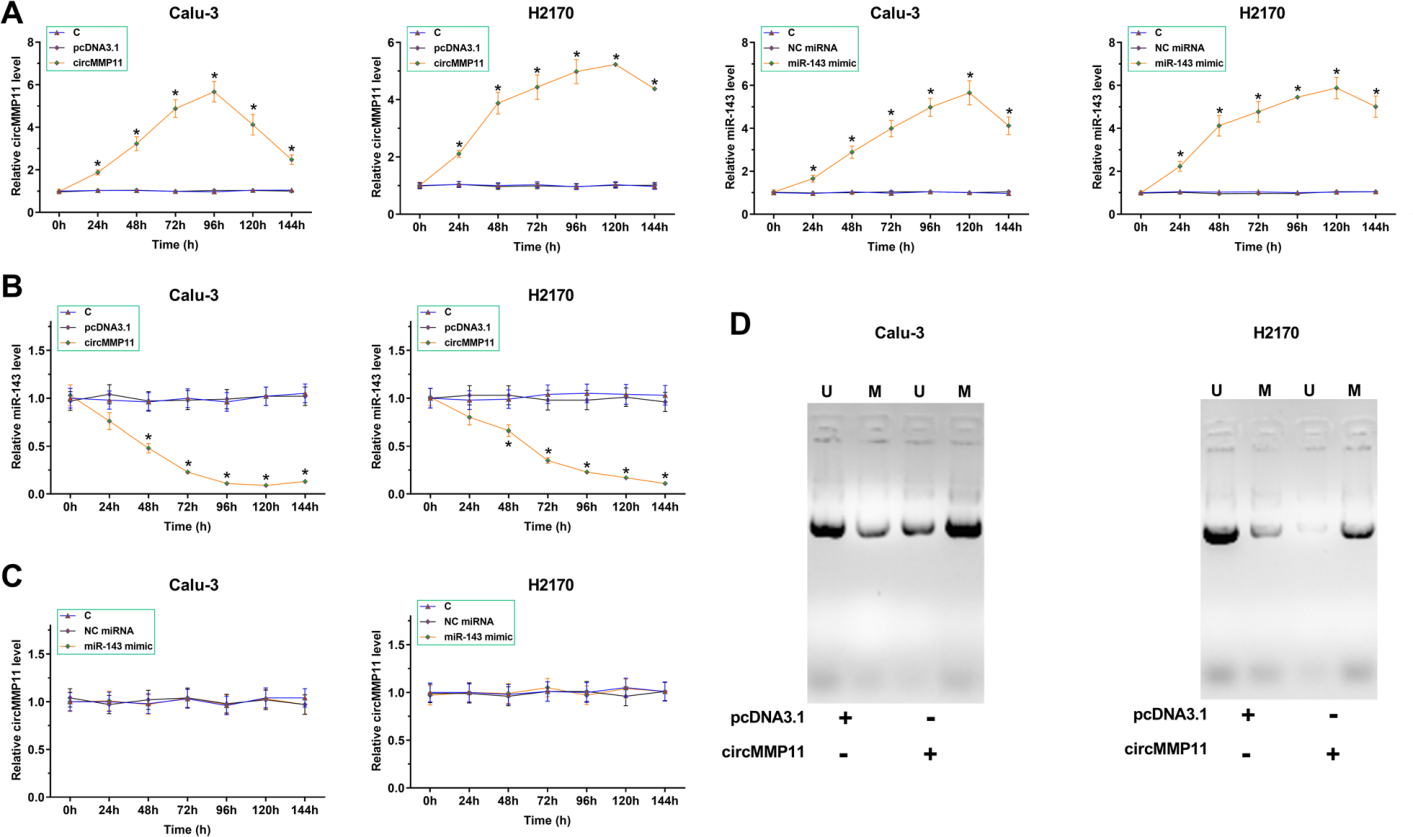
CircMMP11 overexpression decreased miR-143 expression via methylation. To study the crosstalk between circMMP11 and miR-143, Calu-3 and H2170 cells were transfected with either circMMP11 expression vector or miR-143 mimic. Overexpression levels of circMMP11 and miR-143 were monitored every 24 h until 144 h (**A**). The effect of circMMP11 expression vector transfection on miR-143 expression (**B**) and the effect of miR-143 mimic transfection on circMMP11 expression (**C**) were analyzed by RT-qPCRs. The effect of circMMP11 on miR-143 gene methylation was analyzed by MSP (**D**). U, unmethylaion; M, methylation. *p < 0.05

**Fig. 4:**
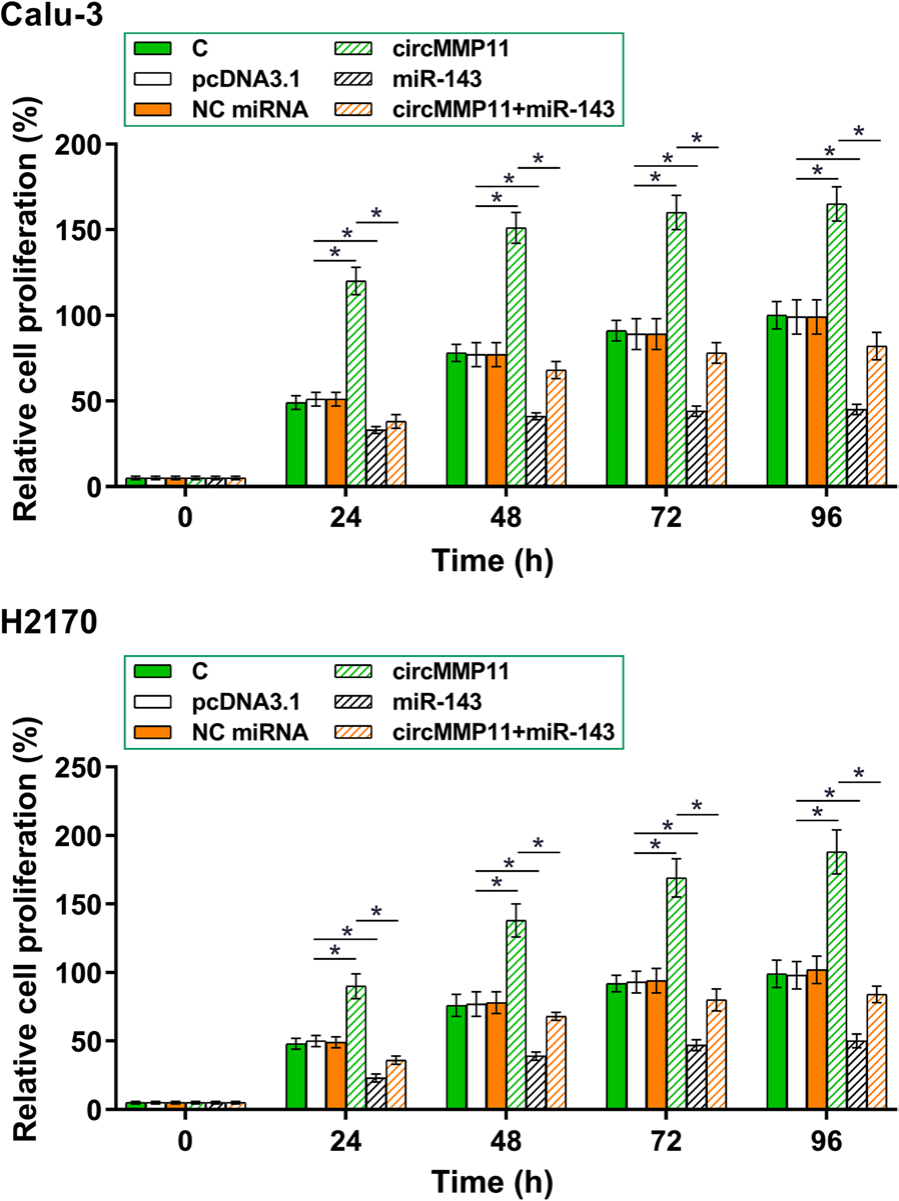
CircMMP11 overexpression increased the proliferation of Calu-3 and H2170 cells via miR-143. CCK-8 assay was performed to analyze the roles of circMMP11 and miR-143 in the proliferation of both Calu-3 and H2170 cells. Cell proliferation was monitored every 24 h until 96 h by measuring the OD values at 450 nm. *p < 0.05

To study the crosstalk between circMMP11 and miR-143, Calu-3 and H2170 cells were transfected with either circMMP11 expression vector or miR-143 mimic. Overexpression levels of circMMP11 and miR-143 were monitored every 24 h until 144 h. It was observed that circMMP11 and miR-143 were significantly overexpressed between 24 and 144 h in both cell lines (Fig. 3A, *p* < 0.05). Interestingly, circMMP11 overexpression decreased miR-143 expression (Fig. 3B, *p* < 0.05) while miR-143 overexpression failed to significantly affect circMMP11 expression (Fig. 4C). The effect of circMMP11 on miR-143 gene methylation was analyzed by MSP. Compared to cells transfected with empty vector, cells transfected with circMMP11 expression vector showed increased methylation (Fig. 3D).

### CircMMP11 overexpression increased Calu-3 and H2170 cell proliferation via miR-143

CCK-8 assay was performed to analyze the roles of circMMP11 and miR-143 in the proliferation of both Calu-3 and H2170 cells. CircMMP11 overexpression significantly increased cell proliferation while miR-143 overexpression decreased cell proliferation. Moreover, co-transfection experiments showed that miR-143 overexpression reversed the enhancing effect of circMMP11 overexpression on cell proliferation (Fig. 4, *p* < 0.05).

## Discussion

In this study, we explored the participation of circMMP11 and miR-143 in NSCLC and analyzed their crosstalk. We found that circMMP11 was overexpressed in NSCLC while miR-143 was underexpressed in NSCLC. In addition, circMMP11 may downregulate miR-143 via methylation to increase cancer cell proliferation.

CircMMP11 has been reported to serve as a miRNA sponge in breast cancer [14]. CircMMP11 is overexpressed in breast cancer to upregulate matrix metalloproteinase-11 (MMP-11) by sponging miR-1204 and thereby increase cancer cell metastasis and growth, suggesting the role of circMMP11 as an oncogenic circRNA in breast cancer [14]. However, the role of circMMP11 in other malignancies is still unclear. We found that circMMP11 was overexpressed in NSCLC and circMMP11 overexpression increased NSCLC cell proliferation. Therefore, circMMP11 is also likely an oncogenic circRNA in NSCLC and circMMP11 downregulation in tumor tissues may serve as a potential target to treat NSCLC. However, how to specifically regulate circMMP11 expression only in tumors but not in normal tissues is challenging in clinical practices.

Despite the advances in the treatment of NSCLC, survival of NSCLC patients is still unsatisfactory, mainly owing to the lack of sensitive diagnostic biomarkers and cures for metastatic tumors. Unfortunately, this situation is unlikely to be changed in the near future. In this study, we showed that circMMP11 was significantly correlated with poor survival of NSCLC patients. Therefore, monitoring circMMP11 expression may help the prognosis of NSCLC patients, thereby guiding to determine the treatment approaches to improve patients’ survival. However, more studies are needed to further analyze the prognostic value of circMMP11 for NSCLC.

MiR-143 has been characterized as a tumor suppressor in many cancers, including NSCLC [15, 16]. For instance, miR-143 is under-expressed in NSCLC and targets epidermal growth factor receptors to suppress NSCLC cell proliferation [16]. Consistently, our study confirmed miR-143 downregulation in NSCLC and its inhibitory effects on cell proliferation. Based on our knowledge, the upstream regulators of miR-143 remain unclear. The study showed that circMMP11 overexpression in NSCLC may downregulate miR-143 by increasing miR-143 gene methylation. However, the methylation factors involved in this process remain to be further identified. It is also known that miR-143 could interact with multiple circRNAs, such as circ-FOXM1 and circFOXO3, to participate in cancer biology [17, 18]. Therefore, the interactions between miR-143 and other circRNAs should also be explored in future studies.

In this study, we characterized circMMP11 as a critical player in NSCLC cell proliferation and demonstrated that circMMP11 might be targeted to treat NSCLC to suppress tumor growth. However, clinical trials are needed to analyze the clinical values of circMMP11 for NSCLC. In addition, this study only enrolled 66 Han Chinese patients. Therefore, our conclusion should be further confirmed in the future with more patients from different ethnic groups.

In conclusion, circMMP11 is overexpressed in NSCLC and it may increase cancer cell proliferation by downregulating miR-143 via methylation.

## Data Availability

The datasets used and/or analyzed during the current study are available from the corresponding author on reasonable request.

## Abbreviations

NSCLC: Non-small cell lung cancer
circRNAs: Circular RNAs
